# Design and Synthesis of Fluoro Analogues of Vitamin D

**DOI:** 10.3390/ijms22158191

**Published:** 2021-07-30

**Authors:** Fumihiro Kawagoe, Sayuri Mototani, Atsushi Kittaka

**Affiliations:** Faculty of Pharmaceutical Sciences, Teikyo University, 2-11-1 Kaga, Itabashi, Tokyo 173-8605, Japan; fkawagoe@pharm.teikyo-u.ac.jp (F.K.); 19dy10003vu@stu.teikyo-u.ac.jp (S.M.)

**Keywords:** synthesis, fluorine, vitamin D_3_, metabolite, A-ring, CD-ring, side-chain

## Abstract

The discovery of a large variety of functions of vitamin D_3_ and its metabolites has led to the design and synthesis of a vast amount of vitamin D_3_ analogues in order to increase the potency and reduce toxicity. The introduction of highly electronegative fluorine atom(s) into vitamin D_3_ skeletons alters their physical and chemical properties. To date, many fluorinated vitamin D_3_ analogues have been designed and synthesized. This review summarizes the molecular structures of fluoro-containing vitamin D_3_ analogues and their synthetic methodologies.

## 1. Introduction

**Scheme 1:**
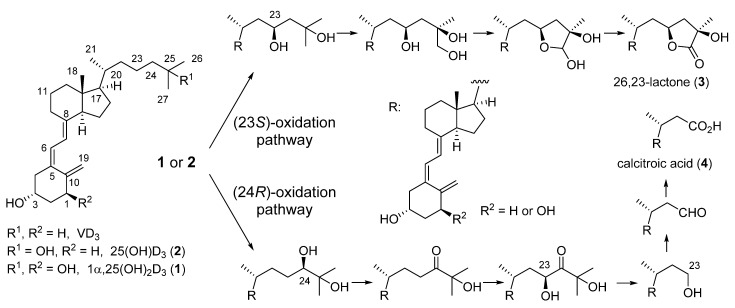
Deactivation metabolic pathways of 25(OH)D_3_ (**2**) and 1α,25(OH)_2_D_3_ (**1**).

Fluorine is one of the halogens, known as a small and the most electronegative element. Fluorine substitution offers a variety of advantages, such as changing the pKa and dipole moment of the molecule, improving the chemical or metabolic stability, and enhancing the binding affinity to the target protein. Furthermore, it has a small atomic radius, similar to that of a hydrogen atom. Due to their unique properties, fluorine atoms have been incorporated into many drugs, drug candidates, and agricultural chemicals [1,2,3,4,5,6,7,8,9,10,11,12]. The contribution of fluorine to drug development and medicinal chemistry, and the life science as well as material science fields, is widely recognized around the world [13,14,15,16,17]. Many scientists have been engaged in the practical synthesis of organofluorine compounds, including fluorinated vitamin D_3_ analogues. Vitamin D_3_ (VD_3_) is a fat-soluble vitamin whose biological function depends on metabolic activation by CYP enzymes [18]. Bioactivation of VD_3_ requires sequential oxidation steps at C-25 and C-1 catalyzed by vitamin D 25-hydroxylase (CYP2R1) and 25-hydroxyvitamin D_3_-1α-hydroxylase (CYP27B1), respectively. The resulting B-secosterol, 1α,25-dihydroxyvitamin D_3_ [1α,25(OH)_2_D_3_ (**1**)], is the fully active and hormonal form of VD_3_. Moreover, 1α,25(OH)_2_D_3_ (**1**) and 25-hydroxyvitamin D_3_ [25(OH)D_3_ (**2**)] are degraded via hydroxylation at C23 or C24 catalyzed by 1α,25-dihydroxyvitamin D_3_-24-hydroxylase (CYP24A1) [19]. C23 hydroxylation and subsequent three-step oxidation lead to vitamin D_3_-26,23-lactone (**3**). On the other hand, C24 hydroxylation and subsequent five-step oxidation lead to calcitroic acid (**4**) (Scheme 1) [19,20].

For slowing or preventing the biological degradation of the VD_3_ side chain, replacing C-H with C-F bond(s) at appropriate positions should prolong their half-life in vivo, since a C-F bond is stronger than a C-H bond chemically. The introduction of fluorine atoms into VD_3_ analogues can also alter electron distribution, which can confer lower pKa at the hydroxy group(s), change the dipole moment, and influence the conformation because of their marked electron-withdrawing properties. Because of these unique properties, both academic institutions and industries have designed and synthesized numerous fluorinated VD_3_ analogues, similarly to other bioactive compounds with fluorine atoms. Most of them show fluorination at the main metabolic site(s) and/or neighboring hydroxy group of the VD_3_ molecule, namely either an A-ring or a side-chain moiety or both. This review introduces fluorinated VD_3_ analogues and their synthetic methodologies, including some basic biological activities.

## 2. A-Ring Fluorinated VD_3_ Analogues

On the VD_3_ A-ring, 1α-hydroxylation is the final and essential step to produce the hormonal form of VD_3_ from 25(OH)D_3_ (**2**), and 1α,25(OH)_2_D_3_ (**1**) exerts a range of physiological activities by binding to the vitamin D receptor (VDR). The A-ring moiety is anchored in the VDR ligand-binding pocket through four hydrogen-bonding interactions, i.e., the 1α-hydroxy group to Ser233 and Arg270, and the 3β-hydroxy group to Tyr143 and Ser274 [21]. As expected, based on the importance of the A-ring moiety, many A-ring fluorinated VD_3_ analogues have been synthesized and evaluated regarding their biological activities.

### 2.1. 1-Fluorinated VD_3_ Analogues

**Scheme 2:**
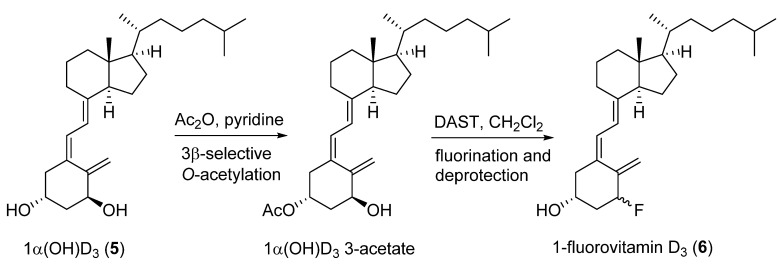
DeLuca’s direct fluorination approach to 1-fluorovitamin D_3_ (**6**).

DeLuca and coworkers described the first synthesis of 1-fluoro-VD_3_ for the purpose of studying the possibility of using it as a kind of VD_3_ antagonist against 1α-hydroxylase in 1979 [22]. They prepared 1α-hydroxyvitamin D_3_-3-acetate by the selective acetylation of 1α-hydroxyvitamin D_3_ [1α(OH)D_3_ (**5**)] and used it as a starting material. The fluorination step was achieved by *N*,*N*-(diethylamino)sulfur trifluoride (DAST) to afford 1-fluorovitamin D_3_ (**6**) (Scheme 2). The authors did not assign its C1 configuration in the report. The biological evaluation revealed that **6** demonstrated a relative preference for stimulating bone calcium mobilization with respect to intestinal calcium transport after metabolism in vivo, and **6** was a weak agonist that could not be used as an anti-vitamin D agent.

**Scheme 3:**
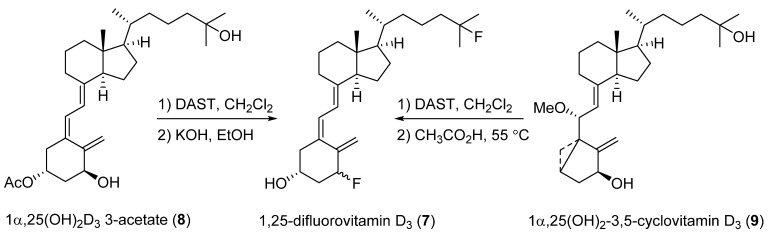
Direct C1 and C25 fluorination approach to 1α,25-difluorovitamin D_3_ (**7**).

Next, DeLuca et al. designed and synthesized 1α,25-difluorovitamin D_3_ (**7**) in 1981 [23]. As shown in Scheme 3, it was synthesized from either 1α,25(OH)_2_D_3_ 3-acetate (**8**) or 1α,25(OH)_2_-3,5-cyclovitamin D_3_ (**9**) using DAST as a fluorination reagent. The authors explained that the stereochemistry at C1 was 1α in the report, but they revised it to 1β in 1984 [24].

The authors evaluated the biological properties of 1α,25-difluorovitamin D_3_ (**7**) and demonstrated that it had essentially no vitamin D activity. These results strongly supported the idea that C1 and C25 hydroxylation are essential aspects of vitamin D function. Initially, since both the C1 and C25 positions of VD_3_ were blocked with fluorine atoms, it might have shown the anti-vitamin D activity of 25-hydroxylation of VD_3_ in vivo. However, **7** did not exhibit the expected inhibitory activity against the 25-hydroxylation of VD_3_.

**Scheme 4:**
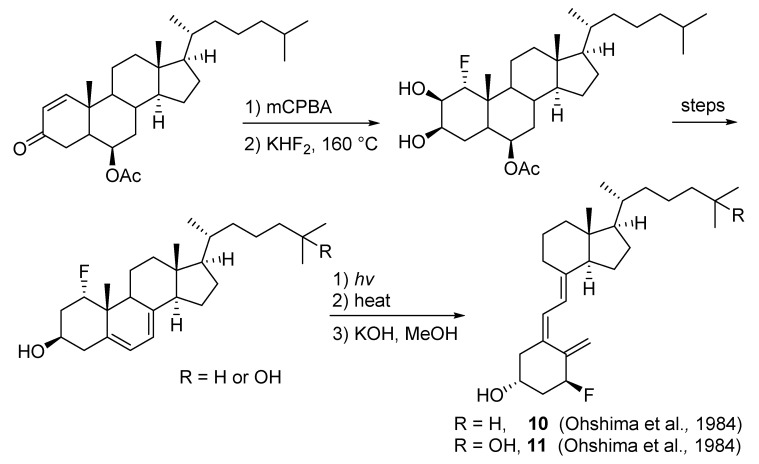
Synthesis of 1α-fluoro-VD_3_ (**10**) and 1α-fluoro-25(OH)D_3_ (**11**) from sterols.

In 1984, 1α-fluorovitamin D_3_ (**10**) and 1α-fluoro-25(OH)D_3_ (**11**) were synthesized utilizing a steroid A-ring epoxide as the starting material in order to confirm the stereochemistry at C1 (Scheme 4) [24,25].

A comparison of the spectral data of the report [24] and previous ones [22,23] revealed that the reported 1α-fluorovitamin D_3_ in 1979 [22] and 1α,25-difluorovitamin D_3_ in 1981 [23] were, in fact, 1β-fluorovitamin D_3_ and 1β,25-difluorovitamin D_3_, respectively.

On the other hand, although 1α-fluoro-25(OH)D_3_ (**11**) showed no stimulation of intestinal calcium transport or bone calcium mobilization activities at a dosage level of 1.3 μg, its binding affinity to chick intestine VDR was 30 times greater than that of 25(OH)D_3_.

**Scheme 5:**
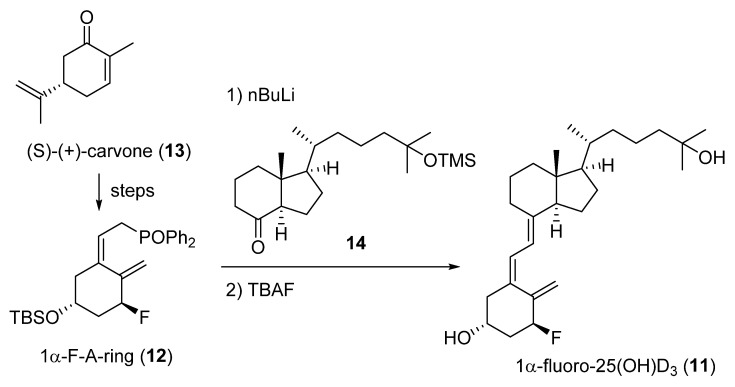
Uskoković’s approach to 1α-fluoro-25(OH)D_3_ (**11**) using the Wittig–Horner reaction.

**Scheme 6:**
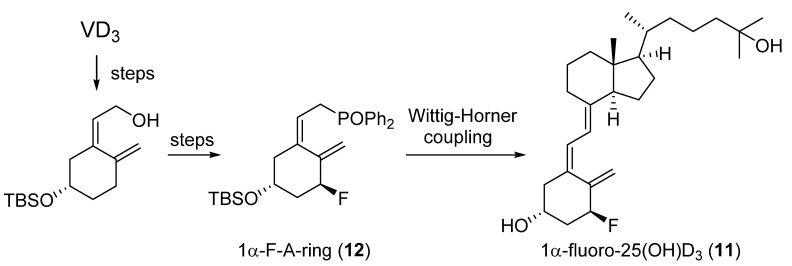
Alternative convergent synthetic approach to 1α-fluoro-25(OH)D_3_ (**11**) using the A-ring moiety (**12**), available from the A-ring of VD_3_.

A convergent synthetic route to 1-fluoro-VD_3_ analogues was described by Uskoković and coworkers using an A-ring key fragment, a 1α-fluorinated A-ring precursor (**12**), which was prepared from (*S*)-(+)-carvone (**13**), in 1990 (17 steps in 4%) (Scheme 5) [26], as well as an A ring from VD_3_ in 1991 (12 steps) (Scheme 6) [27]. A lithium anion of the A-ring phosphine oxide (**12**) underwent a Wittig–Horner coupling reaction with the 8-keto-CD-ring (**14**) to afford the desired 1α-fluoro-25(OH)D_3_ (**11**).

**Scheme 7:**
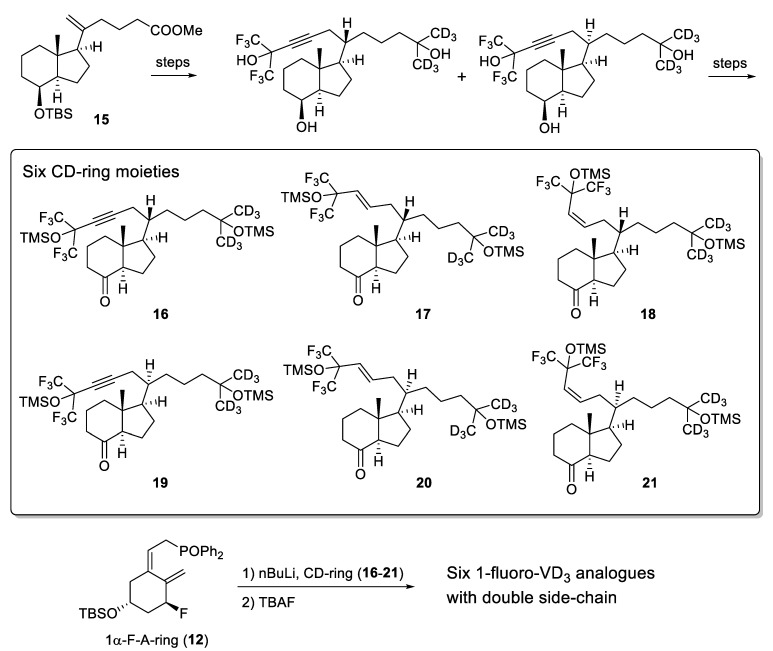
Various 1α-fluoro-VD_3_ analogues with six different double side chains were synthesized using the Wittig–Horner olefination at the coupling stage with the 1α-fluoro-A-ring part (**12**).

Later, Uskoković’s group utilized the 1α-fluoro-A-ring (**12**) to synthesize six 1α-fluoro-VD_3_ analogues with two different side chains at C20 (Gemini analogues). They prepared six 8-keto-CD-rings (**16–21**) starting from the methyl ester (**15**) and coupled it with **12** under basic conditions (Scheme 7) [28]. The anticancer activity of these compounds was tested, but 1α-fluorination did not effectively promote the activity.

**Scheme 8:**
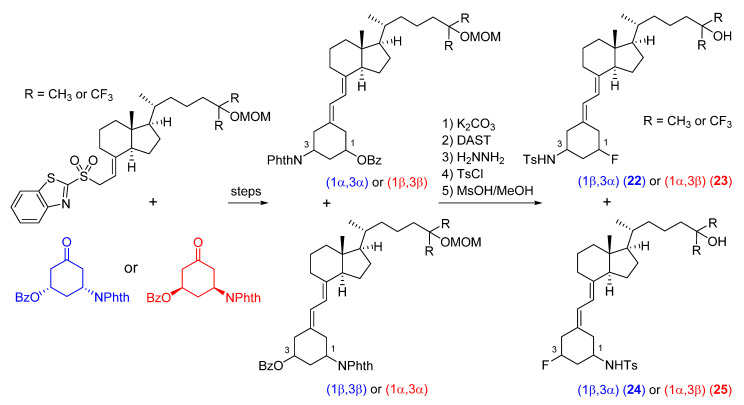
Synthesis of 1- and 3-fluoro-19-norvitamin D_3_ analogues (**22**–**25**) using direct deoxyfluorination reactions.

In 2019, Uesugi and colleagues published the first synthesis of 1-fluoro-19-norVD_3_ analogues [29]. They constructed 1-hydroxy-19-norvitamin D_3_ structures with a modified Julia olefination method [30], and the direct deoxyfluorination of C1 yielded the corresponding 1-fluorinated analogues (**22**,**23**). In this reaction sequence, shown in Scheme 8, they also obtained 3-fluoro-19-norVD_3_ analogues (**24**,**25**). Some of these analogues were poor VDR binders but showed potent sterol regulatory element-binding protein (SREBP) inhibitory activity via inducing SREBP cleavage-activating protein (SCAP) degradation [29].

### 2.2. 2-Fluorinated VD_3_ Analogues

**Scheme 9:**
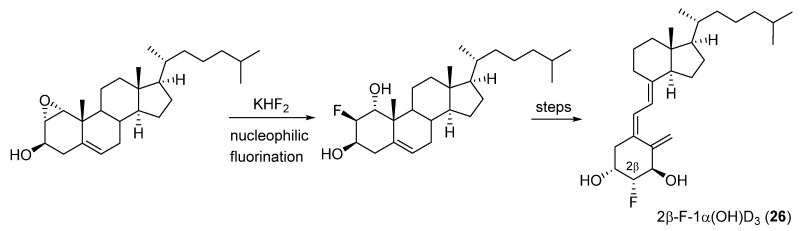
Ikekawa’s approach to 2β-fluoro-1α(OH)D_3_ (**26**) via 2β-fluorination of A-ring epoxide using a nucleophilic fluorination reaction.

Several 2-fluorinated VD_3_ analogues have been reported to date, because fluorine substitution at this position can change the A-ring conformation and pKa value of the neighboring 1α- and 3β-hydroxy groups. The first synthesis of 2β-fluoro-VD_3_ was reported by Ikekawa and coworkers in 1980, in which nucleophilic fluorination using KHF_2_ to 1,2α-epoxycholesterol gave 2β-fluoro-1α(OH)D_3_ (**26**), (Scheme 9) [31]. It was noted that the biological activity of **26** was increased [32].

**Scheme 10:**
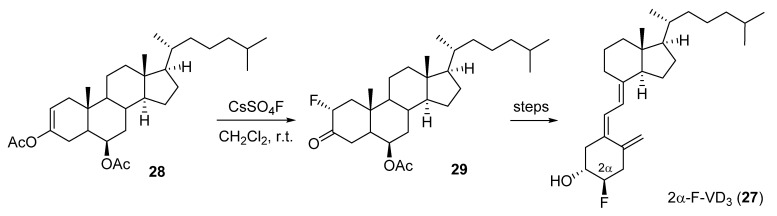
Introduction of the 2α-fluoro group to VD_3_ using an electrophilic fluorination reaction.

Next, 2α-fluorovitamin D_3_ (**27**) was also synthesized, and its biological activity was tested in 1986 [32]. Electrophilic 2α-fluorination of 3,6β-diacetoxycholest-2-ene (**28**) using CsSO_4_F gave 2α-fluoroketone (**29**). To construct the B-secosteroidal structure, conventional photochemical conversion and subsequent thermal isomerization were applied (Scheme 10) [32]. The biological effects of **27** on intestinal calcium transport and bone calcium mobilization as well as the serum calcium concentration at a dosage level of 500 ng for rats were measured, and the activities were found to be essentially equivalent to those of VD_3_ itself.

**Scheme 11:**
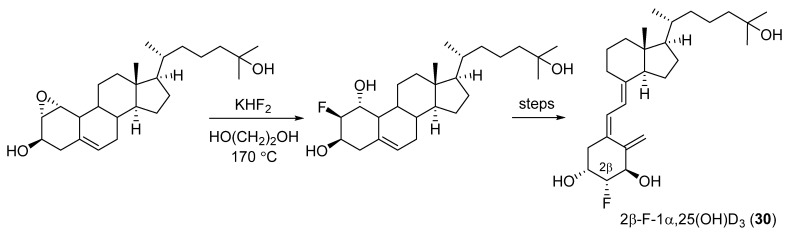
Synthesis of 2β-fluoro-1α,25-(OH)_2_D_3_ (**30**).

The synthesis and biological activities of 2β-fluoro-1α,25-dihydroxyvitamin D_3_ (**30**) were reported by Scheddin et al. in 1998 [33]. The synthetic route was similar to Ikekawa’s [31], as shown in Scheme 11, and the biological evaluation revealed that the synthetic 2β-fluoro-1α,25-(OH)_2_D_3_ (**30**) exhibited greater potency in vitro, for example, six-times higher affinity for VDR, nearly identical affinity for the vitamin D-binding protein (DBP), and 90-times higher antiproliferative activity toward C3H10T1/2 cells under serum-containing conditions, as well as five-times greater adipogenesis inhibitory activity than the natural hormone 1α,25(OH)_2_D_3_ (**1**).

**Scheme 12:**
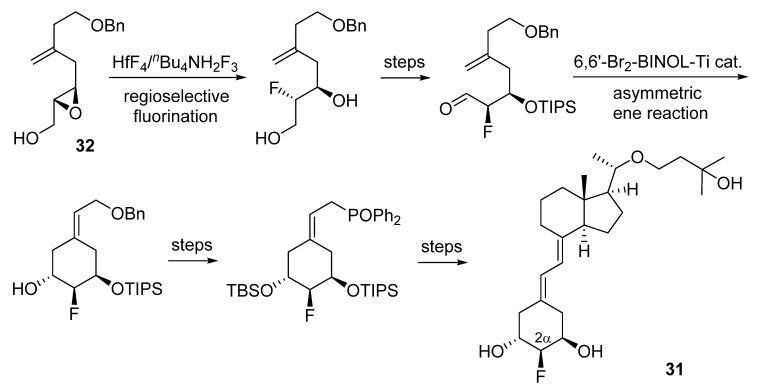
Mikami’s group synthesized 2α-fluoro-19-normaxacalcitol (**31**) using a convergent coupling method between the 2α-fluoro-A-ring part, prepared from regio- and stereoselective fluorination to **32**, followed by asymmetric catalytic carbonyl-ene cyclization, and the appropriate 8-keto-CD-ring.

The catalytic asymmetric stereoselective synthesis of the A-ring precursor of the 19-nor type 2α-fluorovitamin D_3_ analogue (**31**) and its synthesis were reported by Mikami et al. [34,35]. The regio- and stereo-selective 2α-fluorination was achieved via a ring opening reaction of chiral epoxide (**32**) mediated by HfF_4_/Bu_4_NH_2_F_3_, the asymmetric catalytic carbonyl-ene cyclization was used to construct the 6-membered A-ring precursor, and the subsequent coupling reaction with the CD ring afforded 2α-fluoro-19-normaxacalcitol (**31**) (Scheme 12). This 2α-fluoro-22-oxa-19-nor analogue (**31**) had very low DBP-binding affinity but four-times stronger VDR-binding potency than its 22-oxa-19-nor counterpart and also showed significant transactivation activity [34]. It was also shown that **31** was highly effective in inhibiting metastatic tumor growth in vivo without toxicity in terms of hypercalcemia and weight loss [35].

**Scheme 13:**
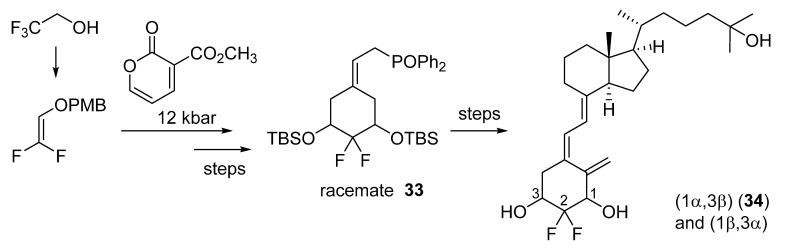
Posner’s synthetic route to 2,2-difluoro-1α,25-dihydroxyvitamin D_3_ (**34**) from 2,2-difluoro-A-ring (**33**) via an inverse-electron-demand Diels–Alder reaction between a pyrone diene and difluorovinyl ether.

Posner’s group showed the first example of synthesis of the 2,2-difluorovitamin D_3_ analogue in 2002 [36]. They synthesized the racemic 2,2-difluoro substituted A-ring phosphine oxide (**33**) from trifluoroethanol (7% in 13 steps). A coupling reaction of **33** with the 8-keto-CD-ring and subsequent deprotection yielded the 2,2-difluoro-1,25-dihydroxyvitamin D_3_ analogues in the ratio of 5:1 (1α,3β (**34**):1β,3α) (Scheme 13). The diastereomers were separated with reversed-phase HPLC to afford the target 2,2-difluoro-1α,25(OH)_2_D_3_ (**34**). Biological evaluation revealed that **34** exhibited antiproliferative activity similar to that of 1α,25(OH)_2_D_3_ (**1**) and was 2-3 times more transcriptionally active than **1** in rat osteosarcoma cells, even though the human VDR-binding affinity was 9.6% relative to that of **1**. Compound **34** showed strong calcemic activity in vivo.

### 2.3. 3-Fluorinated VD_3_ Analogues

The C3 position of VD_3_ has a β-hydroxy group, and substitution of the hydroxy with a fluorine atom is of fundamental interest. There are several reports of replacing the C3-hydroxy group with a fluorine atom in order to demonstrate the expected positive effects on biological activity.

**Scheme 14:**
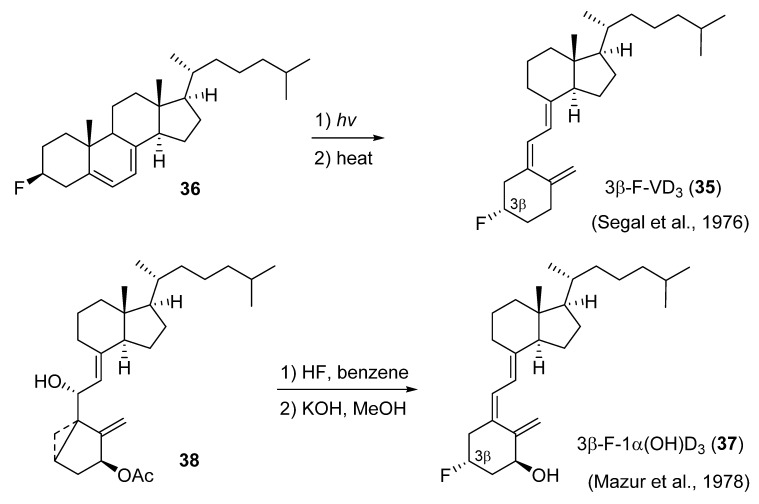
Segal’s and Mazur’s 3β-fluoro-3-deoxyvitamin D_3_ syntheses.

The synthesis of 3β-fluoro-3-deoxyvitamin D_3_ (**35**) from 3β-fluorocholesta-5,7-diene (**36**) via photochemical transformation followed by thermal isomerization was described by Segal et al. in 1976 [37]. It was found that 3β-fluoro-3-deoxyvitamin D_3_ (**35**) had an antirachitic effect analogous to VD_3_. On the other hand, in 1978, Mazur and coworkers designed and synthesized 3β-fluoro-3-deoxy-1α-hydroxyvitamin D_3_ (**37**) to elicit VD_3_ activity. The 3β-fluoro group was constructed from (6*R*)-hydroxy-3,5-cyclovitamin D_3_ (**38**) by treatment with HF (Scheme 14) [38]. The biological activities of the newly synthesized analogue were tested, and the fluoro-analogue (**37**) could actively induce the formation of a calcium-binding protein and stimulate intestinal calcium absorption in rachitic chicks.

**Scheme 15:**
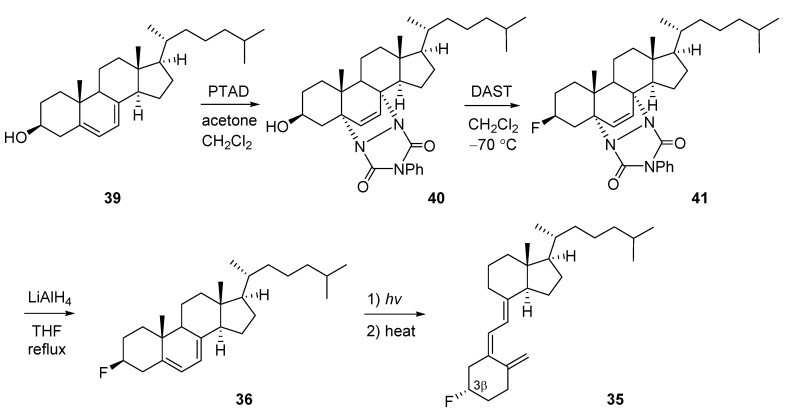
Synthesis of 3β-fluoro-3-deoxyvitamin D_3_ (**35**) starting from 7-dehydrocholesterol (**39**).

Later, in 1985, Kumar and coworkers reported the synthetic route to 3β-fluoro-3-deoxyvitamin D_3_ (**35**) starting from 7-dehydrocholesterol (**39**) via 3-deoxy-3-fluoro-7-dehydrocholesterol (**36**) [39]. The B-ring protected PTAD (4-phenyl-1,2,4-triazoline-3,5-dione) adduct (**40**) was reacted with DAST to yield a fluorinated product (**41**), and subsequent deprotection of **41** gave 3-deoxy-3-fluoro-7-dehydrocholesterol (**36**) (Scheme 15). To investigate the influence of the replacement of the C3-hydroxy group with fluorine, they compared the biological activities of VD_3_, 3-deoxyvitamin D_3_, and 3β-fluoro-3-deoxyvitamin D_3_ (**35**) and revealed that **35** was less active than VD_3_ and more active than 3-deoxyvitamin D_3_ in terms of intestinal calcium transport and bone calcium mobilization in vivo.

As mentioned in Section 2.1, Uesugi and colleagues reported the first synthesis of 3-fluoro-19-norVD_3_ analogues and evaluated their SREBP inhibitory activity vs. VDR activity (see Scheme 8) [29].

### 2.4. 4-Fluorinated VD_3_ Analogues

**Scheme 16:**
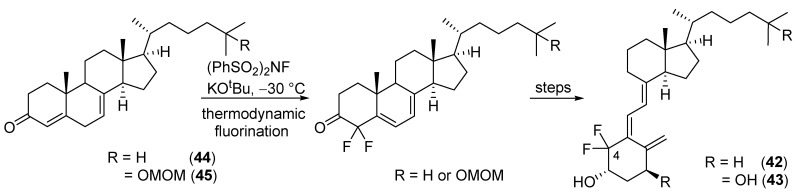
Yamada’s approach to 4,4-difluorovitamin D_3_ (**42**,**43**) using a thermodynamic fluorination reaction.

Yamada and coworkers reported 4,4-difluorovitamin D_3_ (**42**) and 4,4-difluoro-1α,25(OH)_2_D_3_ (**43**) in an A-ring conformational study in 1999, and these analogues were synthesized starting with enones (**44**,**45**), which were constructed from ergosterol, respectively [40]. The difluorination step was achieved by electrophilic fluorination under thermodynamic conditions (Scheme 16). The binding affinity of 4,4-difluoro-1α,25(OH)_2_D_3_ (**43**) for VDR was only ca. 1% of that of the natural hormone, 1α,25(OH)_2_D_3_ (**1**).

## 3. Introduction of a Fluorine Atom into the Triene Part of VD_3_

### 3.1. 19-Fluorinated VD_3_ Analogues

**Scheme 17:**
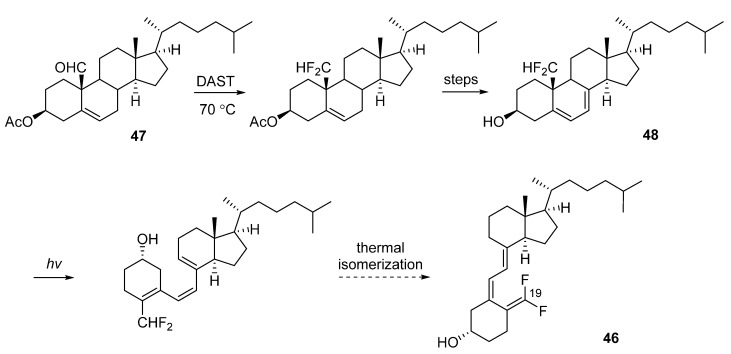
Mazur’s trial to synthesize 19,19-difluorovitamin D_3_ (**46**).

In 1980, the first attempt to synthesize 19,19-difluorovitamin D_3_ (**46**) starting from 19-oxocholesteryl acetate (**47**) via photoirradiation and thermal isomerization reactions using diene (**48**) failed, because the final thermal isomerization step by [1,7]-sigmatropic rearrangement did not proceed in the presence of the difluoromethyl group at the C10 position (Scheme 17) [41].

**Scheme 18:**
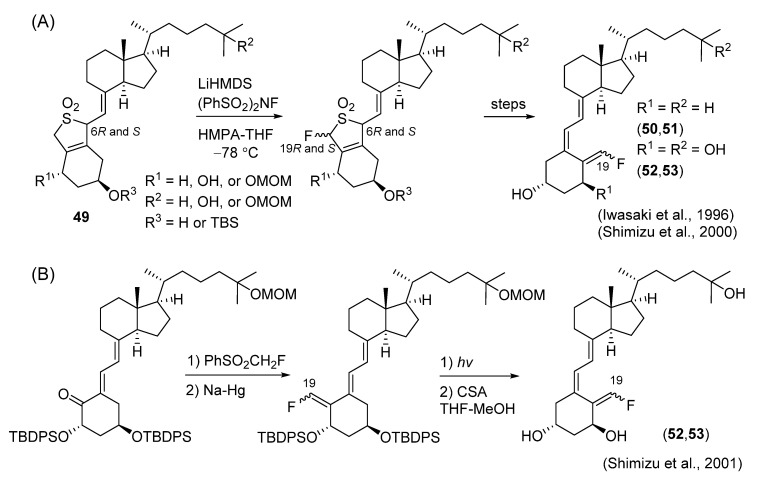
Yamada’s approach to (10*E*)- and (10*Z*)-19-fluorovitamin D_3_ (**50**,**51**) in (**A**) and (10*E*)- and (10*Z*)-19-fluoro-1α,25(OH)_2_D_3_ (**52**,**53**) analogues in (**A**,**B**).

Later, in 1996, Yamada and coworkers showed a novel synthetic route to the first (10*E*)- and (10*Z*)-19-fluorovitamin D_3_ (**50**,**51**) [42]. They prepared VD_3_-SO_2_ adducts (**49**) from VD_3_, and fluorination at the C19 position was achieved by electrophilic fluorination using (PhSO_2_)_2_NF in the presence of LiHMDS (Scheme 18A). In 2000, the same group showed the regioselective introduction of a fluorine atom to the C19 position and synthesized both (10*E*)- and (10*Z*)-19-fluoro-1α,25(OH)_2_D_3_ (**52**,**53**) [43]. The binding affinity of (10*Z*)-19-fluoro-1α,25(OH)_2_D_3_ for VDR was ca. 10% compared with that of 1α,25(OH)_2_D_3_ (**1**). The alternative synthetic route to **52** and **53** from a 10-oxo-19-norVD_3_ derivative was also established by the same group in 2001 (Scheme 18B) [44].

### 3.2. 6- and 7-Fluorinated VD_3_ Analogues

**Scheme 19:**
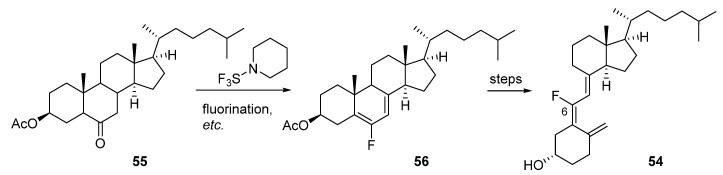
Synthesis of 6-fluorovitamin D_3_ (**54**) using piperidinosulfur trifluoride as a fluorination reagent.

The synthesis of 6-fluorovitamin D_3_ (**54**) was described by Dauben et al. in 1985, and the synthetic route started from 6-oxo-cholestanyl acetate (**55**) [45]. The key intermediate, 6-fluoro-7-dehydrocholesteryl acetate (**56**), was synthesized by allowing **55** to react with piperidinosulfur trifluoride in the presence of sulfuric acid. To construct the triene system, 6-fluoro-7-dehydrocholesterol was irradiated, followed by thermal [1,7]-sigmatropic hydrogen rearrangement, to give the desired 6-fluorovitamin D_3_ (**54**) (Scheme 19). The obtained 6-fluorovitamin D_3_ (**54**) was air-sensitive, and decomposition proceeded. The biological profile of the analogue was evaluated in vivo, revealing that **54** had no biological effect on either intestinal calcium absorption or bone calcium mobilization. However, it significantly inhibited both VD_3_- and 1α,25(OH)_2_D_3_-mediated intestinal calcium absorption through a direct interaction with VDR [46].

**Scheme 20:**
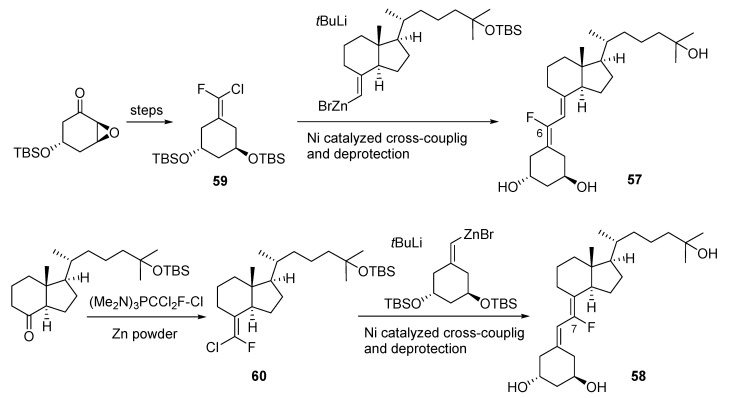
Efficient introduction of the C6- and C7-fluorovinyl unit to the A-ring or CD-ring followed by construction of the diene structures using a Ni-catalyzed cross-coupling reaction.

Furthermore, 6-fluoro-1α,25-dihydroxy-19-norvitamin D_3_ (**57**) and 7-fluoro-1α,25-dihydroxy-19-norvitamin D_3_ (**58**) were synthesized by the Teijin research group in 2004 [47]. Takenouchi et al. prepared a C6-fluorinated A-ring (**59**) and C7-fluorinated CD-ring (**60**), respectively. A Ni-catalyzed cross-coupling reaction with each CD-ring- or A-ring-activated alkene counterpart was used to construct the diene structures of **57** and **58** (Scheme 20). These compounds possessed 10–70% VDR binding affinity of that of 1α,25(OH)_2_D_3_ and potential activity to induce HL-60 cell differentiation.

## 4. CD-Ring Fluorinated VD_3_ Analogues: 11-Fluorinated VD_3_ Analogues

**Scheme 21:**
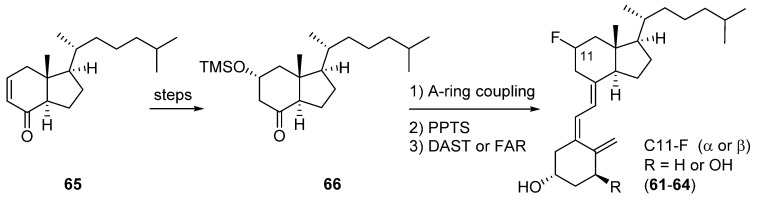
Stereoselective hydroxylation, coupling with the A-ring precursor, and subsequent fluorination at the C11 position.

To our knowledge, only one report has been published on the synthesis of CD-ring fluoro-VD_3_ analogues. In 1994, De Clercq and coworkers designed and synthesized 11α- and 11β-fluorovitamin D_3_ analogues (**61**–**64**) with the aim of inducing a conformational change from *s*-*trans* to *s*-*cis* at the C6-C7 single bond via expected hydrogen bond formation between the C11-F and C1α-OH groups [48]. Enone (**65**), readily available from Grundmann’s ketone, was used as a starting material. Epoxidation of **65**, followed by reductive opening with lithium dimethylcuprate, gave the C11α-OH functional group (**66**). After coupling with A-ring phosphine oxides, the C11α-OH group was treated with *N*-(2-chloro-1,1,2-trifluoroethyl)diethylamine (FAR) to give C11α-F and C11β-F isomers as well as an elimination product at a ratio of 3:1:1 (Scheme 21). NMR spectra analyses of the 11-fluoro-1α(OH)D_3_ showed that all analogues had a large *J*_6-7_ coupling constant, reflecting an almost exclusive *s-trans* extended geometry without intramolecular hydrogen bonding between C11-F and 1α-OH.

## 5. Side-Chain Fluorinated VD_3_ Analogues

The CYP24A1 pathway is well-known as the deactivation pathway of both 25(OH)D_3_ (**2**) and 1α,25(OH)_2_D_3_ (**1**) [19]. Varieties of side-chain-fluorinated VD_3_ analogues have been actively synthesized because of the expected slower catabolism resulting from the presence of fluorine atoms at the oxidation site or adjacent area.

### 5.1. 22-Fluorinated VD_3_ Analogues

**Scheme 22:**
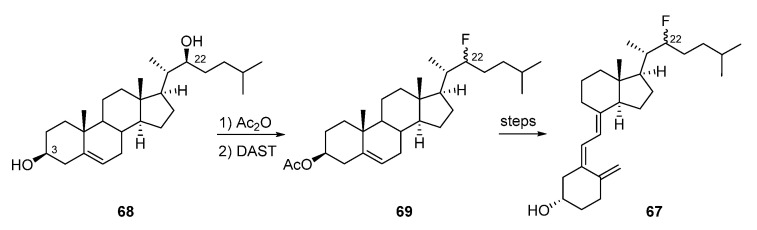
Synthesis of 22-fluorovitamin D_3_ (**67**) from (22*S*)-22-hydroxycholesterol (**68**).

In 1986, Kumar and coworkers described the synthesis of 22-fluorovitamin D_3_ (**67**) starting from (22*S*)-cholest-5-ene-3β,22-diol (**68**) [49]. Selective protection of the C3 hydroxy group as an acetate, followed by fluorination at the C22 position by DAST, gave 22-fluorocholest-5-en-3β-acetate (**69**). After forming the C5-7 diene unit in the B ring, photolysis and thermal isomerization yielded the target 22-fluorovitamin D_3_ (**67**) without assigning C22 stereochemistry (Scheme 22). They tested the biological activities of the analogue in vitro and in vivo, referring to the potency of intestinal calcium transport, serum calcium level, calcium-binding protein induction, plasma vitamin D-binding protein (DBP), and VDR affinities, and concluded that the introduction of a fluorine atom to C22 resulted in the compound, with weak biological activities and poor binding to DBP compared with VD_3_ itself.

### 5.2. 23-Fluorinated VD_3_ Analogues

**Scheme 23:**
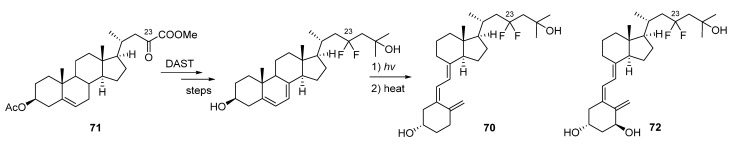
Synthesis of 23,23-difluoro-25(OH)D_3_ (**70**) using α-ketoester (**71**) as a key intermediate, and the structure of 23,23-difluoro-1α,25(OH)_2_D_3_ (**72**) is also shown.

As mentioned in the Introduction, the C23 position of VD_3_ is one of the essential metabolic sites of CYP24A1; therefore, C23-fluorinated VD_3_ analogues have been designed and synthesized based on the idea of blocking the oxidative position. Ikekawa and colleagues achieved the first synthesis of a C23-fluoro-VD_3_ analogue, 23,23-difluoro-25(OH)D_3_ (**70**), in 1984 [50]. For the synthesis of **70**, the triene structure was constructed by applying the well-established route through 5,7-diene steroids, and the difluoro unit was introduced using DAST into a reactive α-ketoester (**71**) (Scheme 23).

The same group continued to report 23,23-difluoro-1α,25(OH)_2_D_3_ (**72**) by the enzymatic 1α-hydroxylation of **70** in 1985 [51]. Their biological activities were evaluated, and 23,23-difluoro-25(OH)D_3_ (**70**) was 5-10 times less active than 25(OH)D_3_ in stimulating intestinal calcium transport, bone calcium mobilization, mineralization of rachitic bone, etc., and 23,23-difluoro-1α,25(OH)_2_D_3_ (**72**) was one-seventh as active as 1α,25(OH)_2_D_3_ in binding to VDR.

**Scheme 24:**
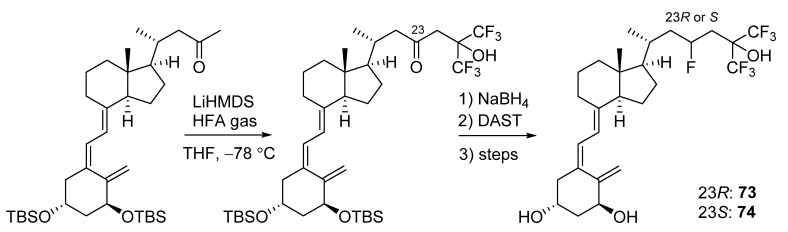
Synthesis of 23,26,26,26,27,27,27-heptafluoro-1α,25(OH)_2_D_3_ using DAST for the seventh fluorine introduction.

Ikeda and coworkers of the Sumitomo research group reported the synthesis of (23*R*)-23,26,26,26,27,27,27-heptafluoro-1α,25(OH)_2_D_3_ (**73**) and its 23*S* isomer (**74**) in 2000 [52]. The starting methyl ketone was available from VD_2_, and a subsequent hexafluoroacetone (HFA) aldol reaction gave a 23-oxo derivative, which was reduced to 23*R*- and 23*S*-secondary alcohols that could be separated and subjected to deoxyfluorination using DAST to afford **73** and **74** (Scheme 24). Both analogues showed higher VDR-binding affinity and HL-60 cell differentiation activity than falecalcitriol.

**Scheme 25:**
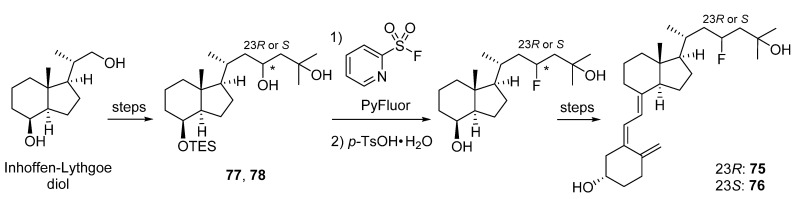
Stereoselective C23-fluorination of 23-hydroxy-CD-rings using PyFluor.

Later, in 2019, our group synthesized (23*R*)-23-fluoro-25(OH)D_3_ (**75**) and its 23*S*-isomer (**76**) starting from the Inhoffen–Lythgoe diol via the key intermediate 23-hydroxy-CD- rings (**77**,**78**) (Scheme 25) [53]. The preliminary biological evaluation revealed that the 23*S*-isomer (**76**) showed higher resistance to CYP24A1 metabolism than its 23*R*-isomer (**75**).

### 5.3. 24-Fluorinated VD_3_ Analogues

**Scheme 26:**
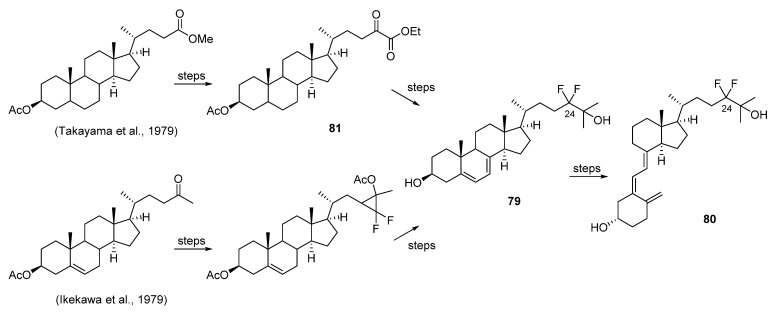
Takayama’s and Kobayashi-Ikekawa’s synthetic routes to 24,24-difluoro-25(OH)D_3_ (**80**).

Since oxidation of the hydroxy function at the C24 position catalyzed by CYP24A1 is one of the important pathways to deactivate both 25(OH)D_3_ and 1α,25(OH)_2_D_3_, developing practical methods to construct the C24-fluoro unit on the VD_3_ skeleton has been pursued since 1979. The first synthesis of the 24,24-difluorovitamin D_3_ analogue was reported independently by Takayama’s group [54] and Kobayashi-Ikekawa’s group [55]. Both synthetic routes involved the key intermediate (**79**), and the two groups synthesized the same analogue, 24,24-difluoro-25(OH)D_3_ (**80**). For the construction of the 24,24-difluoro unit, Takayama and coworkers utilized the reaction of α-ketoester (**81**), which was derived from lithocholic acid, with DAST. On the other hand, Kobayashi et al. used the reaction of steroidal enol ether, derived from cholic acid, with difluorocarbene (Scheme 26).

In 1980, Kobayashi-DeLuca’s group subsequently demonstrated that kidney homogenates from the chicken converted 24,24-difluoro-25(OH)D_3_ (**80**) to 24,24-difluoro-1α,25(OH)_2_D_3_ (**82**) [56]. The results of the biological evaluation revealed that **82** and its nonfluorinated counterpart 1α,25(OH)_2_D_3_ (**1**) equipotently stimulate intestinal calcium transport and bone calcium mobilization in vivo, while in another in vitro system, **82** was found to be four times more potent than 1α,25(OH)_2_D_3_ (**1**).

**Scheme 27:**
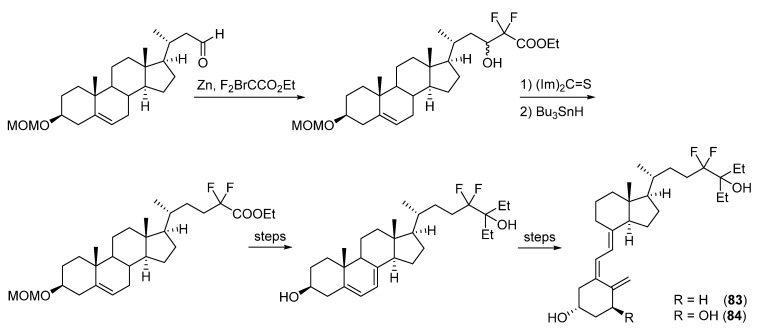
Kumar’s synthetic approach to 24,24-difluorovitamin D homologues (**83**,**84**) using the Reformatsky reaction.

In 1990, Kumar’s group synthesized 24,24-difluoro-25-hydroxy-26,27-dihomovitamin D_3_ (**83**) and its 1α-hydroxy analogue (**84**) from 3β-hydroxy-22,23-dinorcholenic acid using a Reformatsky reaction in their synthetic route (Scheme 27) [57]. Both analogues showed similar biological activities, such as intestinal calcium transport and bone calcium mobilization in vivo, to those of the respective nonfluorinated counterparts and also 25(OH)D_3_ or 1α,25(OH)_2_D_3_.

**Scheme 28:**
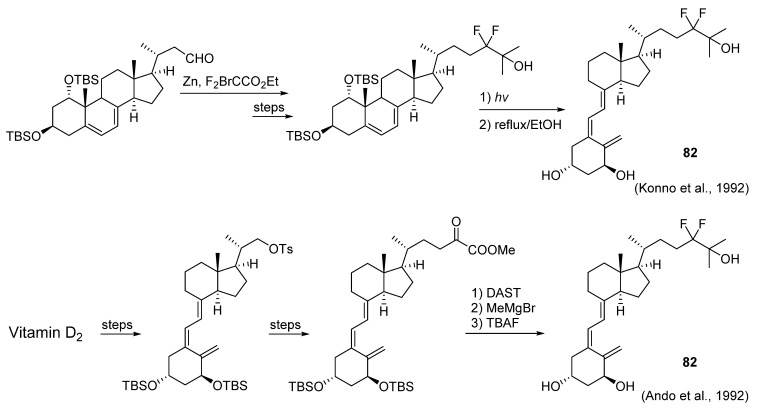
Takayama’s two improved synthetic approaches to 24,24-difluoro-1α,25(OH)_2_D_3_ (**82**).

In 1992, two alternative linear synthetic routes to 24,24-difluoro-1α,25(OH)_2_D_3_ (**82**) were reported by Takayama et al. using the Reformatsky reaction with ethyl bromodifluoroacetate or Horner–Emmons reaction as the key step, respectively (Scheme 28) [58,59]. The starting material of the former was 1α-hydroxydehydroepiandrosterone, with a 3.8% overall yield of **82** [58], and that of the latter was vitamin D_2_, with a 9.3% overall yield of **82** [59].

**Scheme 29:**
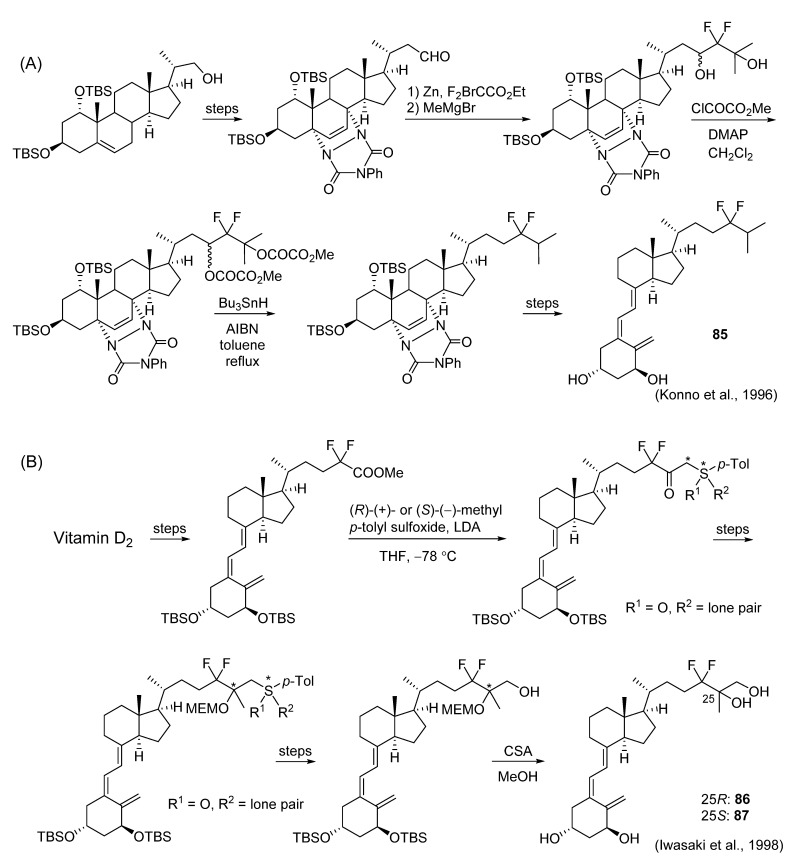
Synthetic routes to 24,24-difluoro-1α(OH)D_3_ (**85**) (**A**) and its CYP24-metabolite (**87**) (**B**) by the Takayama group.

As shown in Scheme 29A, the same group synthesized 24,24-difluoro-1α(OH)D_3_ (**85**) from a steroidal skeleton in 1996 [60]. This compound showed higher activity than 24,24-difluoro-1α,25(OH)_2_D_3_ (**82**) in intestinal calcium absorption. In 1998, Iwasaki-Takayama’s group reported the synthesis of (25*R*)- and (25*S*)-24,24-difluoro-1α,25,26-trihydroxyvitamin D_3_ (**86**,**87**), including the X-ray crystallographic analysis of a synthetic intermediate to determine C25-stereochemistry, and proved that the 25*S*-isomer (**87**) was the main CYP24-metabolite of **82** (Scheme 29B) [61].

**Scheme 30:**
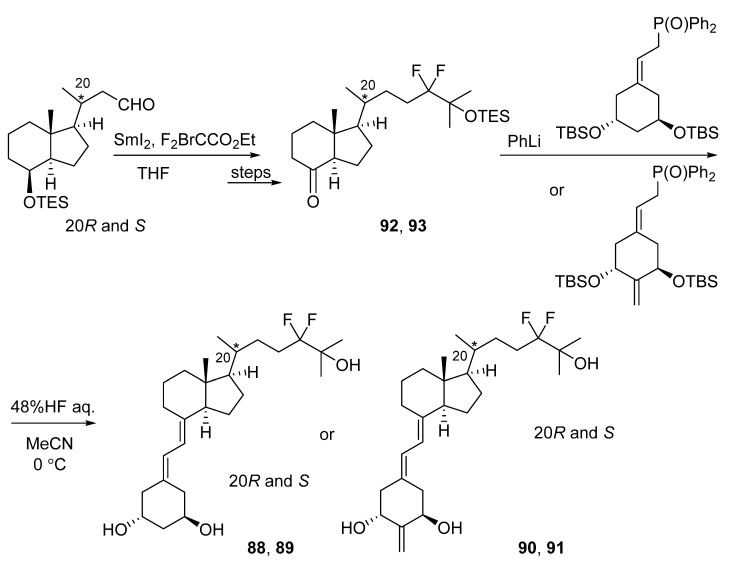
DeLuca’s convergent approach to 24,24-difluoro-1α,25(OH)_2_-19-norVD_3_ (**88**,**89**) and the 2-exomethylene analogues (**90**,**91**) including their 20-*epi* versions using the Wittig–Horner reaction.

The 24,24-difluoro-19-norVD_3_ analogues including 20-*epi*-versions (**88**–**91**) were reported by DeLuca et al. in 2015 [62]. In contrast to the previous synthetic methods starting from steroid skeletons, they demonstrated a convergent method utilizing the Wittig–Horner reaction between 24,24-difluoro-CD-rings (**92**,**93**) and a lithium salt of a phosphine oxide anion from A-ring precursors (Scheme 30). The 20*S*-derivatives showed marked bone-mobilizing activity in vivo; however, 2-methylene substitution was required for such elevated activity in the 20*R* series.

**Scheme 31:**
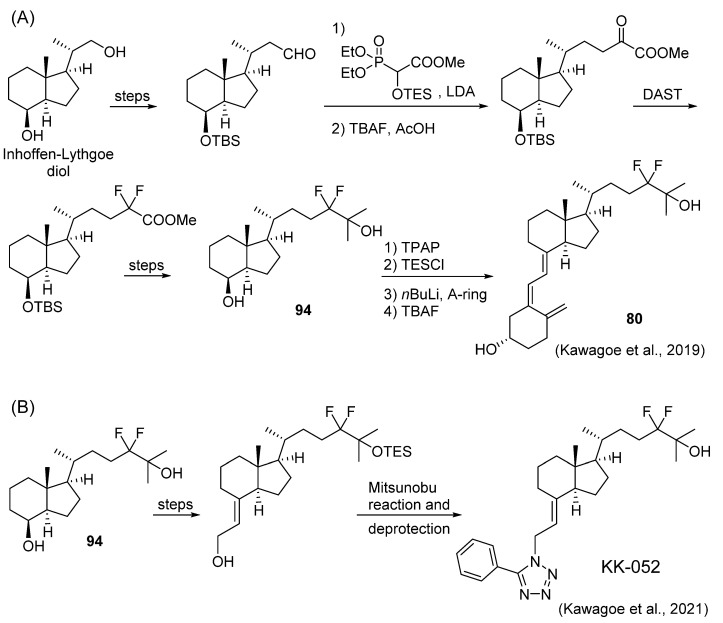
Efficient construction of the 24,24-difluorinated CD-ring unit (**94**) and the subsequent A-ring coupling reaction (**A**). Synthesis of the novel SREBP-specific inhibitor KK-052 (**B**).

In 2019, our group developed the convergent synthesis of 24,24-difluoro-25(OH)D_3_ (**80**) using a coupling reaction between the 24,24-difluoro-CD ring ketone derived from the Inhoffen–Lythgoe diol via **94** and an A-ring phosphine oxide (Scheme 31A) [63]. We subsequently synthesized a novel vitamin D-based VDR-silent SREBP inhibitor, KK-052, from **94** (Scheme 31B) [64].

**Scheme 32:**
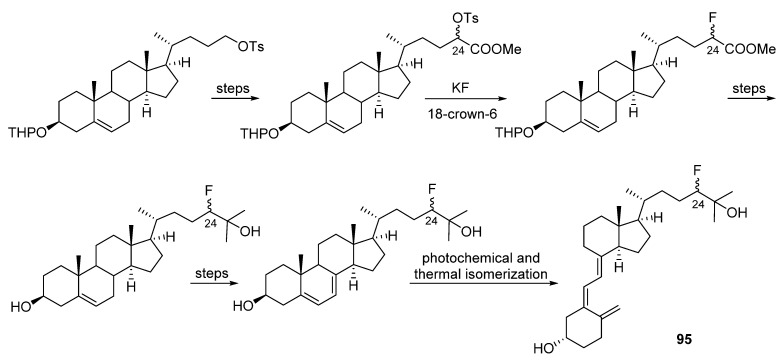
Synthesis of 24-monofluoro-25(OH)D_3_ (**95**) using nucleophilic fluorination as a key step.

Ikekawa’s group reported in 1979 the first synthesis of C24-monofluoro-25(OH)D_3_ (**95**) from cholenic acid without assigning the C24 stereochemistry (Scheme 32) [55].

**Scheme 33:**
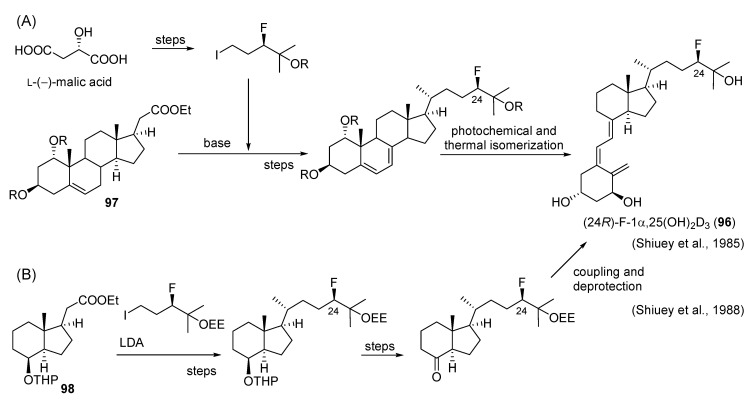
(24*R*)-Fluoro-1α,25(OH)_2_D_3_ (**96**) was synthesized by Uskoković et al. through a linear synthetic route using **97** and optically active L-(‒)-malic acid as a chiral synthon (**A**) and an alternative convergent route starting from a CD-ring part, **98** (**B**).

After this, (24*R*)-24-fluoro-1α,25(OH)_2_D_3_ (**96**) was synthesized by Uskoković’s group through linear (Scheme 33A) and convergent (Scheme 33B) synthetic routes in 1985 [65] and 1988 [66], respectively. In this case, (24*R*)-24-fluoro-1α,25(OH)_2_D_3_ (**96**) showed a longer plasma half-life and higher anti-rachitogenic activity than 1α,25(OH)_2_D_3_ in vivo.

### 5.4. 25-Fluorinated VD_3_ Analogues

**Scheme 34:**
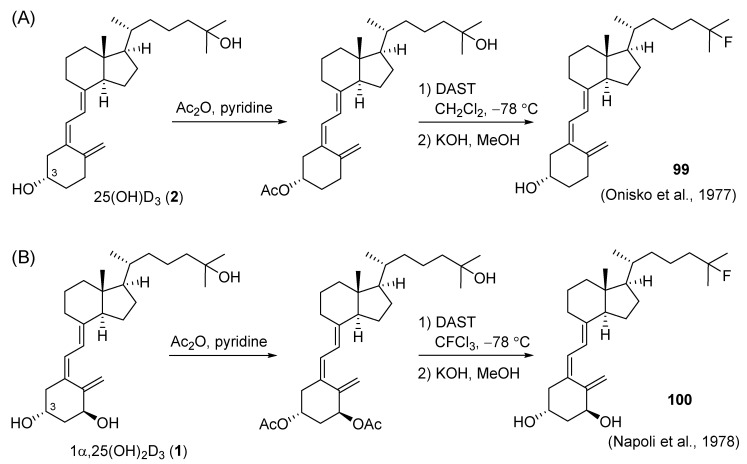
Synthesis of 25-fluoro-VD_3_ analogues via direct C25 fluorination from 25(OH)D_3_ (**A**) and 1α,25(OH)_2_D_3_ (**B**) using DAST as a fluorinating reagent.

The 25-hydroxylation is the initial metabolic conversion of VD_3_ by CYP2R1 or CYP27A1 [18], and 25(OH)D_3_ (**2**) is known as the major circulating metabolite in the human body. The first introduction of fluorine to C25, i.e., the synthesis of 25-fluoro-VD_3_, was reported by DeLuca et al. in 1977 (Scheme 34A) [67]. C3-Selective *O*-acetylation of 25(OH)D_3_ (**2**) and subsequent direct fluorination at the C25 position using DAST, followed by deacetylation, gave 25-fluorovitamin D_3_ (**99**). In 1978, Stern and coworkers synthesized 25-fluoro-1α(OH)D_3_ (**100**) using the same approach (Scheme 34B) [68]. The results of the biological evaluation indicated that 25-fluoro-1α(OH)D_3_ (**100**) was approximately equipotent to 1α(OH)D_3_ (**5**) in VDR binding and stimulating bone resorption in vitro, and the C25-fluoro substituent behaved similarly to hydrogen, without elevating its biological potency.

As described in Section 2.1, DeLuca’s group also synthesized 1,25-difluorovitamin D_3_ in 1981, and this compound was devoid of any biological activity [21].

### 5.5. 26,27-Hexafluorinated VD_3_ Analogues

**Figure 1:**
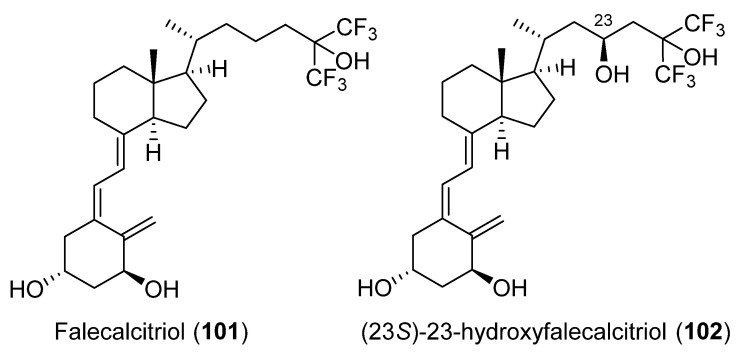
Structures of falecalcitriol (**101**) and its major metabolite (23*S*)-23-hydroxyfalecalcitriol (**102**).

Falecalcitriol (**101**) is 26,26,26,27,27,27-hexafluorinated VD_3_ and has been approved for therapeutic use against secondary hyperparathyroidism in Japan [69,70]. Falecalcitriol (**101**) was ca. 10 times more active in increasing bone calcium mobilization than the natural hormone (**1**) in vivo [71]. Similarly to other fluorinated VD_3_ analogues, it was metabolized more slowly than 1α,25(OH)_2_D_3_ (**1**) by CYP24A1, and interestingly, its major metabolite (23*S*)-23-hydroxyfalecalcitriol (**102**) was equipotent to the parent compound **101** in its biological activity (Figure 1) [72,73,74]. The 23-hydroxylated **102** was specifically glucuronidated by UGT1A3 [75].

**Scheme 35:**
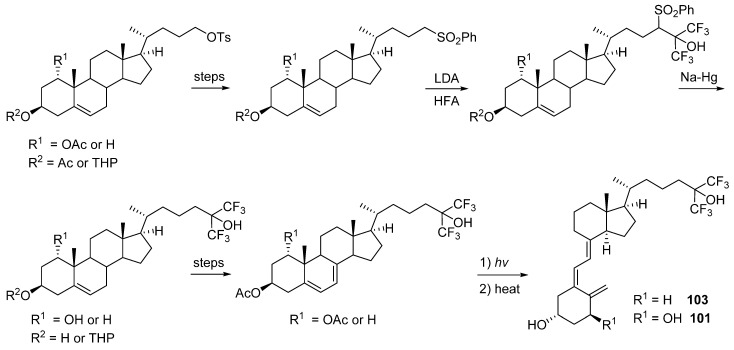
Synthesis of 26,26,26,27,27,27-hexafluoro-25(OH)D_3_ (**103**) and 26,26,26,27,27,27-hexafluoro-1α,25(OH)_2_D_3_ (**101**) using hexafluoroacetone as a fluorine source.

The first synthesis of 26,26,26,27,27,27-hexafluoro-25(OH)D_3_ (**103**) was reported by Kobayashi et al. in 1980 [76], and the same group subsequently synthesized 26,26,26,27,27,27-hexafluoro-1α,25(OH)_2_D_3_ (**101**) in 1982 [77]. In this case, 3β-Tetrahydropyranyloxychol-5-en-24-ol tosylate was used as a starting material, and hexafluoroacetone (HFA) was utilized for construction of the 26,26,26,27,27,27-hexafluoro unit (Scheme 35). The biological evaluation revealed that **101** was approximately 5-10-fold more active than 1α,25(OH)_2_D_3_ (**1**) without inducing severe hypercalcemia.

**Scheme 36:**
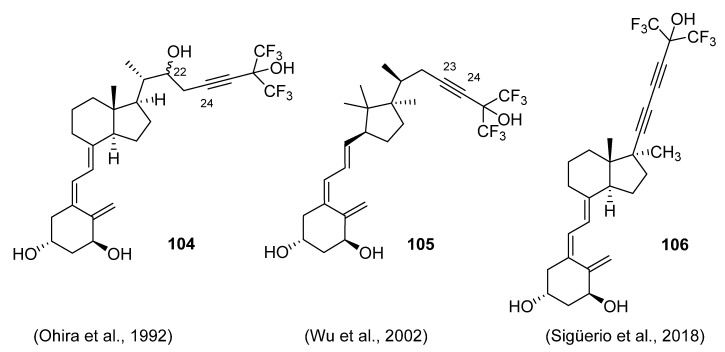
Combination of the hexafluoropropanol unit derived from hexafluoroacetone (HFA) and triple bond(s), which brought conformational rigidity to the side chain.

Using the reaction of acetylides with excess HFA gas as the key step, syntheses of the hexafluoroalkynyl-VD_3_ analogue (**104**), C-*seco*-hexafluoroalkynyl-VD_3_ analogue (**105**), and the previously mentioned two-side-chain analogues **17**-**22** including alkene side chains (Section 2.1) were reported by Ohira et al. in 1992 [78], by Wu et al. in 2002 [79], and by Maehr et al. in 2009 [28], respectively (Scheme 36). Compound **104** had a potent inducing effect on the differentiation of cancer cells, with little calcium mobilization activity. Compound **105** showed comparable VDR-binding affinity to the natural hormone **1** and had strong antiproliferative activity against four cancer cell lines in vitro, with 1% calcemic activity compared with **1** in vivo. More recently, Sigüeiro et al. synthesized C22-diyne analogues with a C17-methyl group, including the hexafluoropropanol unit at the terminal (**106**), which showed potent VDR-binding affinity [80].

Hayashi and colleagues described the introduction of the 26,26,26,27,27,27-hexafluoro unit utilizing an aldol reaction [52]. The C23 ketone was treated with HFA in the presence of LiHMDS to afford the HFA adduct (see Scheme 24 in Section 5.2).

**Scheme 37:**
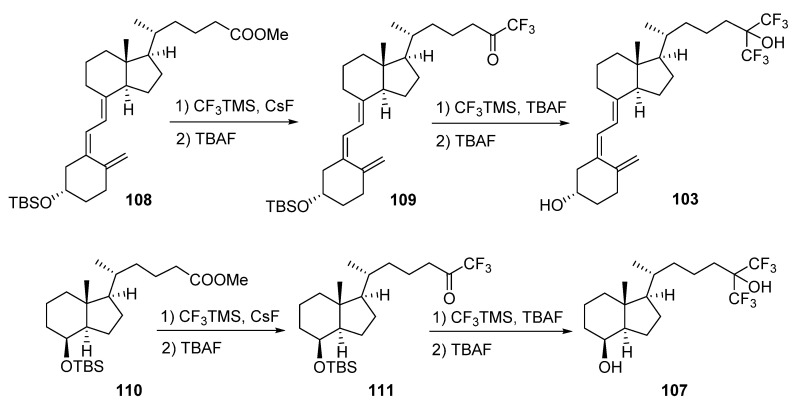
Synthesis of 26,26,26,27,27,27-hexafluoro-25(OH)D_3_ (**103**) and synthetically useful 26,26,26,27,27,27-hexafluoro-CD-ring precursor (**107**) using efficient two-step trifluoromethylation method.

As an alternative path to construct the hexafluoro unit, the nucleophilic trifluoromethylation of methyl esters with Ruppert–Prakash reagent (CF_3_TMS) can be utilized. In our group, 26,26,26,27,27,27-hexafluoro-25(OH)D_3_ (**103**) and 26,26,26,27,27,27-hexafluoro-CD ring (**107**) were synthesized in 2018 starting from the methyl esters (**108**,**110**) through trifluoromethylketones (**109**,**111**) as the intermediates (Scheme 37) [81].

## 6. Summary

This review summarized the historical fluorinated VD_3_ analogues with modification from the A-ring to the end of the side-chain, including their synthetic methods. With the aim of preventing or slowing their activation or degradation by CYPs, the A-ring and side-chain have been mainly focused on, and numerous VD_3_ analogues containing the fluorine atom(s) have been synthesized. Hydroxylation at the C25 and C1α positions of VD_3_ is necessary for the activation process of the molecule by CYP2R1/CYP27A1 and CYP27B1, respectively; therefore, in general, the introduction of fluorine to these positions decreases the biological activity through VDR if compared to non-fluorinated 25(OH)D_3_ or 1α,25(OH)_2_D_3_ as each parent VDR ligand. On the other hand, fluorination at the side chain C23, C24, and C26(27) of VD_3_, where deactivating hydroxylation occurs based on CYP24A1 metabolism, produces strong VDR agonists that have a long half-life in vivo. Among them, falecalcitriol was successfully approved for the treatment of secondary hyperparathyroidism in Japan. Discovery of the new functions of VD_3_ continues; for example, the potent SREBP-inhibitory activity of 25(OH)D_3_ [82] and the new fluorinated analogues with their efficient synthetic methods may contribute to the treatment of patients with VD_3_-function-related disease in the future.
